# Femur Fracture Associated with Knee Brace Wear in the Motocross Athlete: A Report of Two Cases and Review of the Literature

**DOI:** 10.1155/2018/1498541

**Published:** 2018-08-30

**Authors:** Amalie Erwood, Jacob M. Wilson, Andrew M. Schwartz, Mara L. Schenker, Thomas Moore

**Affiliations:** ^1^Emory University School of Medicine, Atlanta, GA, USA; ^2^Department of Orthopaedics, Emory University School of Medicine, Atlanta, GA, USA; ^3^Grady Memorial Hospital, Atlanta, GA, USA

## Abstract

The sport of motocross entails off-road motorcycle racing and is associated with a high incidence of traumatic injury. While prophylactic knee braces are routinely worn, there has been anecdotal concern that brace use is linked to femoral shaft fractures. While this risk remains unreported in the medical literature, preventing this complication has played a role in new commercial knee brace designs. We present two cases in which two motocross riders sustained transverse femoral shaft fractures at the proximal portion of each respective knee brace. The fracture locations measured on anterior-posterior radiograph were 22 and 21.1 cm proximal to the center of the knee, which is also the recommended proximal extent of motocross knee braces. We propose that the rigid knee brace protects the ligamentous knee structures but may focus undue force on the proximal aspect of the brace. New knee brace designs have incorporated features to dissipate the potentially injurious force to prevent femur fracture. While knee braces undoubtedly help prevent ligamentous knee injury, these cases question the safety of standard brace design and highlight the need for further brace development to better protect the patient's bony structures, in addition to the knee joint.

## 1. Introduction

Motocross is an off-road racing sport in which two-wheeled vehicles are ridden over various terrains and jumps. These vehicles can reach high speeds (up to 60 mph in 5 seconds) [1], and there is a high crash rate (94.5% per year for each rider) [2]. Consequently, the sport boasts the second-highest injury rate next to motorcycle racing. This high injury volume has spurred many studies that outline the dangers of this sport to riders, especially in the pediatric population [1–4]. Most commonly, these injuries are to the extremities [5], and Larson et al.'s study [1] demonstrated 89% of injuries that required surgery were orthopedic in nature.

The American Motorcyclist Association (AMA) attempts to mitigate these risks by administering a set of rules annually. The rules dictate mandatory protective equipment that includes helmet with full face coverage, goggles/face shield, long-sleeve jerseys, protective pants, and boots that protect the ankle and foot [6]. However, the mandatory protective gear does not protect against the frequently encountered ligamentous knee injuries [2, 3]. Colburn and Meyer [3] noted that 50% of injuries in their study were ligamentous injuries with knee collateral ligament sprain being one of the most prevalent. As a result, many riders have favored the use of knee braces in an attempt to reduce the incidence of knee ligament damage.

Knee brace use has been associated with diminished incidence of motocross-related soft tissue knee injury [7]. However, there has been speculation that these braces may also serve as stress risers for femoral shaft fractures. Although there are no known published cases of such fractures, injury associated with protective sport equipment is not unprecedented and is especially well-documented in skiing with the phenomenon of “boot top” fractures [8–10].

In this report, we discuss two cases in which motocross riders sustained transverse femoral shaft fractures during competition. Both riders were wearing a knee brace at the time of their injury, and their fractures were found to correlate with the top of their respective braces. We aim to describe the fracture pattern and highlight future work needed to prevent knee brace-associated femur fractures without compromising ligamentous knee protection.

## 2. Case Presentations

### 2.1. Case 1

**Figure 1:**
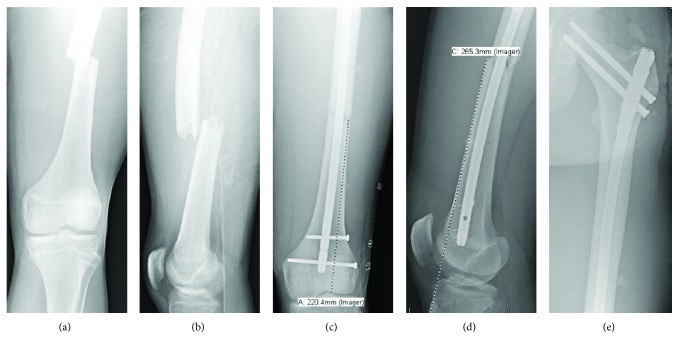
(a) AP radiograph of the left femur and (b) lateral radiograph of the left femur obtained upon patient presentation to the Emergency Department. (c) Postoperative AP radiograph of the left femur demonstrating fracture line 22.0 cm proximal to the center of the knee. (d) Postoperative lateral radiograph of the left femur demonstrating fracture line 26.5 cm proximal to the tibial tuberosity. (e) Postoperative lateral radiograph of the left proximal femur.

A 17-year-old male professional motocross athlete with a history of left tibial spine avulsion fracture and resultant chronic knee flexion contracture presented to the emergency department (ED) status-post motocross injury with isolated left thigh pain. The patient had been wearing a hard-shell, hinged, knee brace measuring approximately 43 cm in length. He reported riding over a jump of approximately 10 feet when his left leg slipped off, pinning and hinging his leg over his knee brace. He was found to have a closed and neurovascularly intact transverse femoral shaft fracture without ecchymosis, skin changes, or open wounds. The deformity measured approximately 26 cm from the tibial tuberosity on clinical exam, and the fracture was 22 cm proximal to the center of the knee as measured on anterior-posterior (AP) radiograph (Figure 1). Per institutional protocol, thin-slice computed tomography (CT) was obtained to rule out femoral neck fracture, and this was negative for fracture [11]. The patient was placed in Buck's traction and prepared for surgical intervention. Anterograde intramedullary nailing of the left femur with a femoral reconstruction nail was performed the next morning. The patient received routine perioperative antibiotic prophylaxis, unrestricted postoperative weightbearing, and one month of chemical deep vein thrombosis (DVT) prophylaxis. The patient had returned to full activity and competitive motocross at one-year follow-up.

### 2.2. Case 2

**Figure 2:**
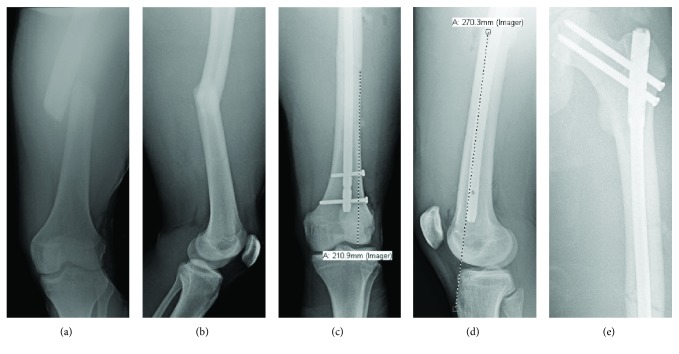
(a) AP radiograph of the left femur and (b) lateral radiograph of the left femur obtained upon patient presentation to the Emergency Department. (c) Postoperative AP radiograph of the left femur demonstrating fracture line 21.1 cm proximal to the center of the knee. (d) Postoperative lateral radiograph of the left femur demonstrating fracture line 27.0 cm proximal to the tibial tuberosity. (e) Postoperative radiograph of the left proximal femur.

A 17-year-old male professional motocross athlete with a history of pediatric left tibial shaft fractures (treated nonoperatively and complicated by painless varus malunion) presented to the ED after crashing his dirt bike. He had been wearing a hard-shell, hinged, knee brace measuring approximately 42 cm in length when he fell on his left side and hyperextended his left leg over the top of his knee brace. The patient complained of isolated left thigh pain. Evaluation of the patient revealed a closed, neurovascularly intact transverse femoral shaft fracture without ecchymosis, skin changes, or open wounds. The deformity was approximately 27.0 cm from the tibial tuberosity on clinical exam and measured 21.1 cm proximal to the center of the knee on AP radiograph (Figure 2). His baseline tibial deformity was unchanged. Again per institutional protocol, thin-slice CT pelvis was obtained to assess for associated femoral neck fracture, and this was negative [11]. The patient was taken to the operating room (OR) the next morning and was treated with an anterograde femoral reconstruction nail with cephalomedullary screws. He received routine perioperative antibiotic prophylaxis, unrestricted postoperative weightbearing, and one month of chemical DVT prophylaxis. At one-year postoperative follow-up, the patient had regained full function and had returned to motocross at his preinjury level.

## 3. Discussion

We describe two cases in which motocross athletes suffered transverse femoral shaft fractures at the proximal edge of their prophylactic knee braces. The fracture locations suggest that while traditional knee braces protect the knee, they may be associated with femur fracture. The patients' knee braces measured approximately 43 and 42 cm in length. The fractures were located approximately 26 and 27.0 cm from the tibial tuberosity and on AP radiograph measured 22 cm and 21.1 cm proximal to the center of the knee. The average market knee brace company recommends that the edges of the knee brace extend “8 inches,” or 20.3 cm above and below the center of the knee to achieve proper fit. Thus, the location of these fractures correlate with half the length of each rider's respective knee braces and the manufacturer's recommended proximal knee brace extent [12].

**Figure 3:**
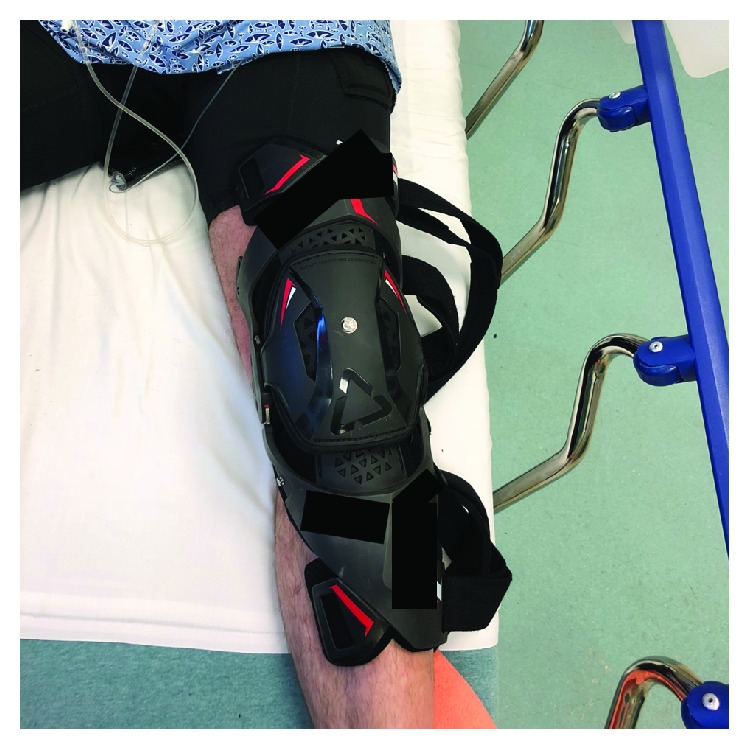
A representative photo of a motocross knee brace. The braces worn by our patients terminated just distal to the site of fracture.

The idea that prophylactic knee bracing may have led to femoral shaft fracture is further supported by the mechanism of injury in our cases. The riders described similar mechanisms where their leg became pinned after slipping off the bike. In both of these injuries, the femur was hyperextended in a 3-point bending nature over the anterior edge of the hard-plastic knee brace. This explains the transverse fracture pattern seen in both cases, as opposed to a spiral fracture pattern often caused by rotational injury (Figure 3).

**Figure 4:**
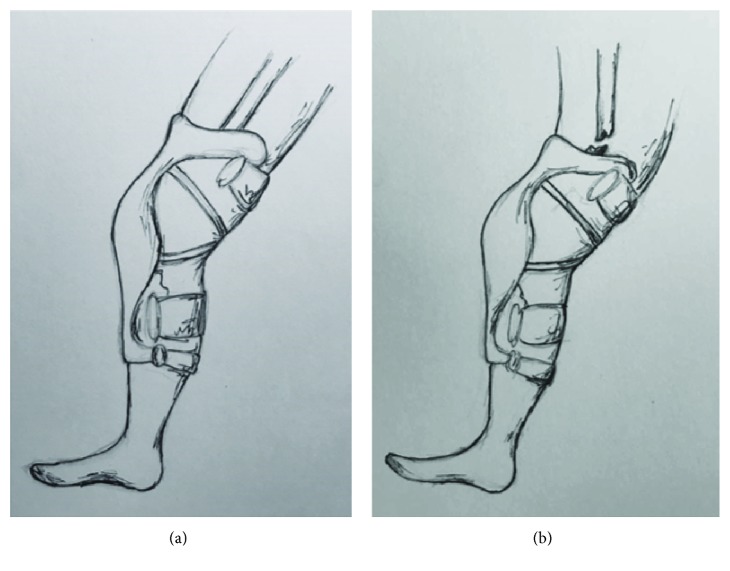
Representative sketch of proposed mechanism of injury. (a) As the leg is subjected to an extension moment, the knee brace extends to its terminal extension point. At this point, further extension force is exerted primarily at the proximal and distal ends of the brace. This force is then absorbed, obligatorily, by the femur at the proximal end of the brace. When this force exceeds the femur's ability to withstand this bending force, it (b) fractures.

Given the clinical history and radiographic findings, we propose that the rigid design of the knee brace protects the knee but may transfer the force proximally, creating a stress riser for cantilever bending. This bending ultimately leads to femoral shaft fracture (Figure 4). This pattern of injury is similar to the “boot top fracture” described in the skiing literature [8–10]. In the 1960s, the phenomenon of the boot top fracture was described as a transverse tibial fracture that occurred when a skier fell forward with heels fixed to the skis, causing a fracture at the proximal edge of the ski boot [10]. Similarly, our riders' legs were bent over the top of their braces, causing a transverse femoral shaft fracture at the proximal edge of the knee brace.

The idea of protective gear causing a specific pattern of injury is further historically supported by a series of specific injury patterns that have resulted from adjustments in skiing equipment. The aforementioned boot top fracture findings led to an alteration of ski boots' safety bindings, and the ski boot was made more flexible in order to reduce the incidence of boot top fractures [10, 13, 14]. Although these adjustments caused a reduced number of overall ankle and tibia fractures, the point of injury moved proximally resulting in an increased number of knee injuries and proximal tibia fractures [15–17]. Additionally, the recent use of carving skis has been associated with a rise in knee injuries, especially in female athletes, when bindings have not been recently adjusted [16, 17].

Although there is a possibility that knee braces predispose motocross athletes to femur fracture, we do not recommend that these athletes discontinue knee brace wear as they have proven effective in protecting against ligamentous knee injury. The incidence of knee ligamentous injury is far greater than that of femur fracture in this cohort, and the brace therefore has a favorable risk-benefit ratio [7]. Gobbi et al. [2] noted 146 (7.8%) cases of knee injury in their 12-year study of motocross injuries. Although this same study noted 171 lower extremity fractures [7], there has been no suggestion to date that a knee brace was associated with the mechanism of fracture [2, 7]. Moreover, Sanders et al. [7] showed prophylactic knee bracing resulted in a clinically significant reduction in the incidence of ACL and MCL injury, while only noting one femoral fracture in the braced group. Contrarily, 79 cases of knee injury were reported including ligamentous knee injury (ACL, MCL, LCL, and PCL tears) meniscal tear, patellar fracture, and patellar tendon tear in the combined braced and nonbraced groups [2, 7].

Despite the paucity of evidence identifying this problem in the medical literature, motocross industry has reacted to anecdotal evidence, and adjustments to knee brace design have already begun. The primary purpose of the motocross knee brace is to prevent knee hyperextension. Consequently, most effective braces are made of a rigid carbon fiber frame. Based on Kennedy et al.'s biomechanical study results [18], using a 3-point bending system, the mean force required to fracture a femur is 4180 N with lateral-to-medial bending and 3780 N for posterior-to-anterior. Bracing companies have recently begun to engineer braces to fail at forces lower than the minimum injury forces needed to fracture both the femur and tibia [18–20]. These engineered sites prevent the femur from absorbing the full impact at the proximal-most portion of the brace.

In conclusion, this series is the first to describe a possible association between the current gold standard prophylactic knee brace design and transverse femur fractures. Although we do not advise against the use of prophylactic knee bracing, this study warrants further evaluation to determine the true incidence of femur fracture related to prophylactic knee bracing. A biomechanical study may lend further support for our proposed mechanism of this fracture pattern and empirically aid the design of safer braces.
